# Concerns, perceived risk, and hesitancy on COVID-19 vaccine: a qualitative exploration among university students living in the West Bank

**DOI:** 10.1017/S0950268823001267

**Published:** 2023-08-07

**Authors:** Guido Veronese, Fayez Mahamid, Dana Bdier

**Affiliations:** 1 University of Milano-Bicocca, Milan, Italy; 2 An-Najah National University, Nablus, Palestine

**Keywords:** COVID-19 pandemic, Palestine, vaccination hesitancy, vaccine risks, vaccination concerns

## Abstract

The current study aimed to explore Palestinian university students’ perceptions and concerns about COVID-19 vaccination hesitancy. Our sample comprised 50 university students selected using snowball sampling techniques from Palestinian universities in the West Bank, Palestine. Thematic content analysis was conducted to identify the main themes of semi-structured interviews with students. The results of the thematic content analysis yielded four main themes: *Students’ perceptions and concerns on COVID-19 vaccinations, perceived risks of vaccination, experiences related to vaccination, and causes of vaccination hesitancy.* Participants expressed concerns and doubts about the vaccine’s safety, showing high hesitancy and scepticism; they also reported different causes for COVID-19 vaccination hesitancy in the Palestinian context, such as the lack of confidence in vaccines, false beliefs about vaccines, and peculiar political instability and conflict of the Palestinian territories enduring a military occupation undermining the health system’s capacity to respond to the COVID-19 outbreak appropriately. Health authorities and policymakers are urgently called to invest in and potentiate awareness campaigns to change the diffuse people’s stereotypes related to the COVID-19 vaccine in the Palestinian territories.

## Introduction

In December 2019, the coronavirus was detected in the city of Wuhan, China, and the Word Health Organization (WHO) officially termed it ‘COVID-19’ [1]. Around the world, the death toll reached over six million recorded units, making the Pandemic one of the most relevant health crises of the century. Thus, a global vaccination campaign rapidly developed new-generation vaccines against COVID-19 to diminish the Pandemic’s impact and overcome it [2]. By the beginning of December 2020, multiple COVID-19 vaccine varieties were authorized and recognized by national health authorities and the World Health Organization (WHO) worldwide [3–6].

Overall, there are many concerns among individuals on the safety of COVID-19 vaccines; most often, people are reluctant or reject COVID-19 vaccine uptake, making vaccine hesitancy a widespread and recognizable global public health [7]. Vaccine hesitancy is characterized by the delay in the vaccines’ acceptance- or blunt refusal- that might reflect the failure or lack of adequate public health messaging, mainly when sceptical individuals do not belong to radical ‘anti-vaxxers’ conspiracy groups [8, 9].

Although vaccine hesitancy was recognized as one of the top 10 public health threats in 2019 by WHO, vaccine acceptance rates were low to medium in several countries. In recent studies, the acceptance of vaccine rates was found to be in Jordan (28.4%), Kuwait (23.6%), Russia (54.9%), the US (56.9%), Poland (56.3%), France (58.9%), and Italy (53.7); the lowest rate of was reported in the Middle East countries [10–13]. In another study that tested vaccine acceptance in different populations, it was found that the lowest COVID-19 vaccine acceptance rates were observed in Hong Kong, ranging from 4.2 to 38%, and in the Democratic Republic of the Congo, corresponding to 15.4% [14].

In the literature, concerns about long-term side effects and unknown future health effects were correlated with COVID-19 vaccine hesitancy [15]. Previous side effects on other routine vaccines, such as the influenza vaccine, can be considered a facilitating factor in vaccine reluctance [16]. Low trust in vaccines, including their safety, efficacy, and importance, contributed to vaccination hesitancy [17, 18]. Lack of confidence in producing and manufacturing vaccines, the pharmaceutical industry, vaccine technology, and public health bodies increased vaccination reluctance [19, 20]. In addition, concerns about the speed of vaccine development of COVID-19 vaccines, vaccines’ incompatibility with religious beliefs, and previously negative healthcare experiences, including racial discrimination, were studied as risk factors for vaccine hesitancy [21–23]. Perception of lower risk of COVID-19 was diminishing the availability of vaccination, while a lack of communication from trusted health providers and community leaders has led to an increase in misinformation [24].

Practical concerns, such as inconvenient vaccine delivery or inaccurate patient contact information, contributed to COVID-19 vaccination reluctance [25, 26]. It diminished the opportunity to be vaccinated in remote or disadvantaged neighbourhoods [16]. In these cases, concerns surrounding fertility, pregnancy, and breastfeeding, and belief in conspiracy theories such as the credibility of COVID-19 or the irrational idea that COVID-19 vaccines might change the DNA’s structure lead to vaccination reluctance [27–29]. Finally, less-educated individuals, younger with lower incomes, and individuals residing in rural areas were associated with COVID-19 vaccine hesitancy [30].

In the present study, because of the Palestinian- Israeli conflict, neither Palestinian individuals nor the state itself has the power to control/secure their borders or to create a country-wide strategy for COVID-19 prevention [31]. In Palestine, by the mid of February 2021, the UN stated that ‘Israel has not ensured that Palestinians under occupation in the West Bank and Gaza will have any near-future access to the vaccines’ [32]. In June 2021, the COVID-19 vaccination rate in Israel was above 60%. In Palestine, below 10%. In order to transfer vaccines from Israel to the Palestinian Authority, the two governments signed an agreement that was characterized as a loan that the Palestinian Authority must pay in the future. However, when the vaccines arrived from Israel, they were found to expire shortly, making the Palestinian Authority cancel the original agreement [33]. When Israel announced the launch of ‘a limited vaccination campaign’ for the occupied territories, they focused on vaccinating tens of thousands of Palestinians who regularly entered Israel and Jewish settlements for work reasons. In contrast, ‘most of the several million Palestinians living under Israeli control in the occupied territories had to wait’ [34, 35].

Regarding the COVID-19 vaccine hesitancy in Palestine, it was revealed that the spread of false rumours, misinformation, and conspiracy theories came from social media. The mistrust towards the vaccines the government purchased was the primary factor contributing to increasing vaccine hesitancy rates among Palestinians [36, 37]. In a study aimed at assessing the willingness of Palestinians to receive a COVID-19 vaccine and their knowledge about vaccines, it was found that about 63% of participants will get a COVID-19 vaccine. Those with good knowledge about the vaccine and its side effects were more willing to get the first dose [38]. In a study carried out with Palestinian healthcare providers, it was found that 37.8% of the participants had intentions to be vaccinated, while 31.5% were undecided, and 30.7% planned to refuse. Younger-age practitioners working in non-governmental settings and with higher COVID-19-related knowledge showed intentions to be vaccinated [39].

In the present study, we set out to answer the following questions in a population of university students: What are the perceived risks of COVID-19 vaccination from the perspective of Palestinian students? How do Palestinians report their experiences related to COVID-19 vaccination? What are the leading causes of COVID-19 vaccination hesitancy from the perspective of Palestinian university students?

## Method

### Participants

Participants in the study were (50) university students selected using convenience and snowball sampling techniques from four leading Palestinian universities in the West Bank, Palestine. Regarding their academic level, respondents comprised postgraduate students (16), graduate students (13), and undergraduate students (21). In total, 27 were females and 23 were males. The ages of participants were between 20 and 52 years (mean age of males = 36.19 years, *SD* = 3.98; mean age of females = 33.09 years, *SD* = 4.51). The study’s inclusion criteria required participants to be: (1) free from mental and neurodevelopmental disorders, (2) Palestinian university students, (3) living in the West Bank of Palestine, and (4) native Arabic speakers.

### Instruments and procedures

The current study’s data were collected in June 2022 using semi-structured interviews and targeted Palestinian university students in the leading academic institutions in the West Bank of Palestine. Participants were selected from four out of eight universities in the West Bank; we selected our sample from these universities as they are spread across all areas in the West Bank of Palestine. Participants were selected using snowball sampling techniques; the local research assistants who received training in qualitative research techniques conducted all semi-structured interviewers with participants. The sample was recruited by contacting the councils of universities and explaining the study’s aims, the planned research procedure, the average number of students we intended to interview, and the selection procedure. Then, a snowball sampling technique was used to identify practitioners in each institute interested in participating in the research and being interviewed. During the interviews, a licenced clinical psychologist was available to provide psychological services to any distressed participant. Participants were provided with contacts in the mental health services to seek help if symptoms arose subsequently to take part in the study. Our study was approved by An-Najah Institutional Review Board (IRB) before collecting the data for this study.

### Data analysis

All interviews were audio-recorded and transcribed in Arabic by a mother-tongue researcher. We also conducted a thematic content analysis of the written transcripts to identify the main themes emerging from the transcripts. Five experts reviewed the authors’ themes; the results showed that the five experts all agreed with the themes.

## Results

The results of thematic content analysis of transcripts yielded four major themes: *Students’ perceptions and concerns on COVID-19 vaccinations, risks of vaccination, experiences related to vaccination, and causes of vaccination hesitancy.*

### Theme one: students’ perceptions and concerns on COVID-19 vaccinations

Vaccines are considered a public health achievement, and the development of COVID-19 vaccines has been described as the top scientific development of 2020; COVID-19 vaccines may save lives by reducing the risk of infection among vaccinated individuals. Our participants showed conflicting responses concerning the role of the vaccine in dealing with the COVID-19 pandemic and reducing infection risk factors. One undergraduate female stated, ‘I believe vaccines protect against infection with the COVID-19 virus and against the side effects associated with the virus’ (22 years old undergraduate female).

Another student doubted the positive results of taking COVID-19 vaccinations; he reported, ‘I think there is no definitive official evidence on the benefits of COVID-19 vaccines’ (22 years old, undergraduate male). One participant added, ‘Nothing is certain about the effectiveness of vaccines, and there is still controversy about the effectiveness and risks associated with vaccines. I believe vaccine manufacturers are the only beneficiaries’ (30 years old postgraduate students). Another participant reported, ‘I do not think that vaccines are necessary; I think that drug manufacturers want to sell as many of these vaccines as possible, and I also think that the number of deaths related to the virus is incorrect’ (22 years old, undergraduate male student). One participant summed up his concern regarding the use of COVID-19 vaccines; he said, ‘I prefer not to use vaccines at present, as there is a need for those vaccines to undergo further studies to prevent any side effects associated with receiving the COVID-19 vaccine’ (20 years old, undergraduate female student).

### Theme two: perceived risks of vaccination

Although many people agreed to receive the vaccine, a high rate of people still believes that vaccines are risky. This could undermine efforts to achieve herd immunity. Therefore understanding people’s general attitude towards vaccination risk is crucial to successfully implementing a large-scale vaccination program. Our participants reported different vaccination supposed risks that may prevent them from receiving vaccination; one participant mentioned, ‘I think that the risk factors for taking vaccinations are related to the possibility of heart attacks’ (21 years old undergraduate female student).

Another participant added, ‘I think the risks associated with vaccines against the COVID-19 virus, that these vaccines can weaken the immune system of vaccinated people’ (28 years old, graduate male student). One participant expressed his opinion on the risks of COVID-19 virus vaccines; he said, ‘vaccines against the COVID-19 virus can cause individuals to suffer serious health problems in the long run. I think vaccines are hazardous, and I do not encourage anyone to take the vaccine’ (29 years old, female graduate student). Another participant added, ‘I do not recommend using any COVID-19 vaccines, as these vaccines are prepared quickly and may have many dangerous side effects’ (51 years old, postgraduate female student).

In the language of another participant explained the risks of COVID-19 vaccination ‘Vaccines against the COVID-19 virus are still new, so the risks associated with taking the vaccine are unknown’ (41 years old, postgraduate female student).

### Theme three: experiences related to vaccination

Developing a safe and efficacious vaccine could be one of the most promising strategies to curtail the virus, save lives and end the COVID-19 pandemic. Our participants reported diverse experiences related to vaccination against the COVID-19 pandemic in Palestine. Indeed, participants expressed more negative experiences related to the COVID-19 vaccines than once. One graduate student said, ‘I know people who died immediately after receiving the COVID-19 vaccine; also, there are people who became allergic after receiving the vaccine’ (46 years old female graduate student).

Another participant added, ‘a relative of mine died after receiving the COVID-19 vaccine, as he developed complications after the vaccine and then died. I also know some people who have had heart infections and other health problems after vaccination’ (24 years old undergraduate female student). One participant disclosed her experiences while he was working as a volunteer in one of the COVID-19 vaccination centres; she said, ‘I worked as a volunteer in one of the vaccination centres, and I know people who faced sleep and digestion problems after receiving the COVID-19 vaccine’ (23 years old undergraduate female students, Al-Quds Open University).

Another participant reported, ‘I did not note any case that faced serious complications after receiving the COVID-19 vaccine; most of the people I know have had minor symptoms such as a mild headache and hand pain because of the injection’ (50 years old, postgraduate male student). Another participant expressed his supportive view of receiving vaccinations; he said, ‘I have received two doses of the COVID-19 vaccine so far, and when I got infected with the virus, my symptoms were very mild; my doctor attributed this to the vaccinations I received’ (24 years old, undergraduate female student). One of the undergraduate participants expressed his experience with COVID-19 vaccines; he reported, ‘Personally, I did not find any benefits of vaccines because the vaccine did not protect me against infection with the virus. I got infected twice with the virus after receiving the vaccine’ (25 years old, undergraduate student).

### Theme four: causes of vaccination hesitancy

Vaccine-hesitant individuals are a heterogeneous group with varying degrees of indecision about specific vaccines or vaccination. Vaccine-hesitant individuals may accept all vaccines but remain concerned; some may refuse or delay some vaccines but accept others; others may refuse all vaccines. Our participants reported different causes for COVID-19 vaccination hesitancy in the Palestinian context. One undergraduate student explained, ‘In my opinion, the main reason behind not receiving COVID-19 vaccines is the lack of confidence in vaccines and the false beliefs that many people hold about vaccines’ (28 years old, undergraduate male student). Another participant expressed, ‘The spread of rumours about the negative effects of COVID-19 vaccines caused many people not to receive vaccines. Unfortunately, most false beliefs about the vaccines came from health providers’ (30 years old, graduate male student). One participant confirmed the role of social media in refusing vaccination; he said, ‘Social media played a negative role in refusing COVID-19 vaccines by publishing an unrealistic picture of the vaccine and its associated complications’ (39 years old, postgraduate student).

Another participant mentioned, ‘People in Palestine became less confident in vaccines when the Palestinian Ministry of Health announced that it had received vaccines from Israel, whose expiry date was approaching’ (37 years old, graduate male student). One participant explained, ‘Many people in Palestine do not trust vaccines because they believe vaccines are a trick aimed at harming them and stealing their lands’ (36 years old, postgraduate female student).

## Discussion

This work explored the perceptions of university students living in Palestine about COVID-19 vaccine safety and their will to get vaccinated. Students in the major Palestinian universities expressed concerns and doubts about the vaccine’s safety, showing high hesitancy and scepticism [40].

Concerns about health consequences and lack of empirical evidence on the vaccine’s short- and long-term adverse effects emerged among the students. The respondents reflected trivial and common-sense opinions, most widespread on social media, rather than reporting information by health authorities and scientific literature [41]. In Palestine, students refer to social media as a means to connect with the external world as, most of the time; they are prevented from travelling because of Israeli military rules [42, 43]. Thus, such instruments played a crucial role in spreading panic about the virus and vaccination during the Pandemic through fake and alarming news [44]. Interviewees seemed to be partially influenced by the extensive anti-vaccine content that frequently is shared across social media, such as Facebook or Twitter platforms, while on the opposite, they used those tools as a source of information and knowledge about vaccination campaigns and their efficacy [45, 46].

Most interviewees were referring to their personal experiences and anecdotal rumours that mirrored the diffuse misinformation and harmful myths about COVID-19 vaccine unsafety [47, 48]. In the recent recalls of students emerged unpleasant memories, adverse symptoms or a sense of unusefulness in being vaccinated. Hence, the interviewees tended not to recommend people adhere to the Palestinian authority vaccination campaign, diminishing the value and the positive outcome of the containment of the widespread virus. A scarce trust in the local health authority or an underestimation of the virus-related health risks was evident [49].

Interviewees reported that in their experience, the efficacy of the vaccination was inconsistent; feeding the false credence that getting vaccinated might mean not contracting the virus anymore. Surprisingly, some of those credences came from medical students who showed low information in their field, an unexpected tendency to vaccine refusal, and a reported reluctance to get vaccinated by the Palestinian health providers themselves [39, 50, 51].

Finally, some of our respondents related their hesitancy to the peculiar political instability and conflict of the Palestinian territories enduring a military occupation undermining the health system’s capacity to respond to the COVID-19 outbreak appropriately [52]. Students referred to a dependency on the Israeli health system regarding virus control and the vaccination campaign [53]. The emerging concern is that the Israeli occupier could use the vaccination campaign and the overall COVID-19 crisis to control and surveillance over Palestinian lives. As a result, people look at vaccines from Israel with fear and suspicion, preferring to adhere to conspiracy myths rather than accepting them as a new technology of the military occupation [36, 54].

Some limitations of the current work must be identified and acknowledged. Our respondents belong to an educated and potentially more aware population. Lower educated and lower socio-economic classes are not represented in this research, limiting the possibility of generalization of our results. However, it provides a fascinating picture of the common sense of the COVID-19 vaccine in Palestine from the perspective of students that we suppose could have been more informed about this topic. A more comprehensive sampling strategy and mixed-method research design are recommendable for the future advancement of this research. A deeper focus on the political and conflict-related antecedents and determinants of vaccine hesitancy and refusal is recommendable for developing knowledge on vaccines and stereotypes in Palestine.

## Conclusion

Our findings revealed vaccine hesitancy among Palestinian students living in the West Bank. Health authorities should invest their efforts in raising awareness and potentiating information channels to fight prejudices and stereotypes in Palestinian society [55]. Working on a trustworthy and effective information campaign should be accompanied by the immediate availability and accessibility of the vaccine in the Palestinian territory that could augment trust and confidence among the population, reducing fear and concerns about the dangers and showing evidence about the benefit of getting vaccinated over catastrophic phantasies, most often even related to the Israeli control over the Palestinians life [32].

To sum up, Palestinians need to be aware of the importance of a vaccine campaign for COVID-19 once they have been given secure and immediate access to the vaccines and all medical resources to battle the Pandemic. Health providers should engage in campaigns to raise awareness about vaccination in the Palestinian context. Also, counsellors, social workers, and mental health providers should conduct interventions to change negative and irrational beliefs towards vaccinations. Moreover, religious leaders, in conjunction with health authorities, can support and coordinate awareness campaigns and mobilize communities for COVID-19-appropriate behaviours and vaccination. They can also provide leadership at the community level to promote COVID-19 prevention activities in Palestine.

## Data Availability

The data that supports the findings of this study are available within the article.
